# Case Report: Multi-cancer early detection utilizing blood-based genomics: Single-institution case series of novel cancer screening

**DOI:** 10.3389/fonc.2025.1637999

**Published:** 2025-08-18

**Authors:** Somya Khare, Jason C. Burton, Francis De Asis, Saron Mekonnen, Mason J. Stewart-McLellan, Diana Potts, Tiffani L. Howard, Nima Nabavizadeh

**Affiliations:** ^1^ Department of Radiation Medicine, Oregon Health & Science University, Portland, OR, United States; ^2^ Cancer Early Detection Advanced Research Center, Knight Cancer Institute, Oregon Health & Science University, Portland, OR, United States; ^3^ Department of Clinical Psychology, Midwestern University, Glendale, AZ, United States; ^4^ Community Outreach and Engagement, Knight Cancer Institute, Oregon Health & Science University, Portland, OR, United States

**Keywords:** cancer early detection, pathfinder, cancer genomics, retrospective study, cancer screening, precision oncology, blood-based screening

## Abstract

**Purpose/Objective(s):**

Cancer screening continues to be a major challenge, with reliable tests only being available for very few cancers. Multi-cancer early detection (MCED) genomic tests are being developed that allow for blood-based screening of multiple cancers simultaneously. The PATHFINDER study was a multi-institution prospective cohort study in healthy participants over the age of 50 years (no cancer history, or history of treated cancer > 3 years prior), investigating the feasibility of the Galleri (GRAIL, LLC) cfDNA methylation MCED blood test. For participants in which the Galleri MCED test revealed methylation signatures indicative of cancer, predicted cancer signal origins were provided to the clinicians to assist with further diagnostic workup. Our institution was the highest accruing site nationally. Here, we describe our institutional test performance and provide informative case vignettes.

**Materials/Methods:**

Under IRB approval, a retrospective chart review of participants enrolled in the PATHFINDER study was performed. Cancer risk factors, outcomes of tests and studies performed due to MCED signal positive, time to diagnostic resolution, and treatment outcomes were obtained from chart-review.

**Results:**

From January 2020 to December 2020, our institution enrolled 1735 participants (26% of total study enrollment), 27 of which returned a signal positive for cancer suspicion (1.6%), and ultimately 12 diagnosed cancers (true positives) for a positive predictive value of 44%. Four of 12 were recurrent cancers in participants more than three years from cancer therapy. There were 15 signal positives without cancer diagnoses (false positives), with one patient receiving extensive work-up for possible uterus, breast or lung cancer origin. Six of 15 false positive results correlated to monoclonal B-cell lymphocytosis (chronic lymphocytic leukemia precursor). During the course of 12-month follow-up for signal negatives, 19 additional participants were diagnosed with a cancer (sensitivity: 39%, specificity: 99.1%).

**Conclusion:**

Our institutional experience demonstrates the feasibility of MCED testing. Additional prospective randomized clinical trials are needed before widespread adoption. The information and data included in this manuscript was previously presented as a poster (e612 Poster Q&A Sessions) at the 2024 American Society for Radiation Oncology Annual Meeting.

## Introduction

Cancer screening continues to be a major challenge, with reliable evidence-based tests only being available for breast, lung, cervical, and colorectal cancer. These screening strategies have made great strides in reducing the cancer burden, morbidity, and mortality of these disease sites; however, in 2023 roughly 60% of the new cancer cases and 60% of cancer-related deaths in the United States are due to types of cancer for which we do not have reliable screening methods (1).

Using the advances of machine learning and high-throughput genomics, Multi-Cancer Early Detection (MCED) tests are being developed that allow for rapid screening of multiple cancer types concurrently through blood tests. These tests are designed on the principle that all tumors have shared biological features with a common set of cellular products accessible through the circulatory system (2). The PATHFINDER study was a prospective return-of results MCED study that enrolled healthy participants over the age of 50 years to assess the safety and feasibility of the Galleri (GRAIL LLC, Menlo Park, CA, USA) MCED cell-free DNA methylation testing for cancer screening. If a cell-free DNA methylation signature was indicative of cancer, up to 2 cancer signal origin(s) informed further clinician diagnostic assessment (3).

Our institution was the highest recruiting center on the PATHFINDER study, enrolling 1735 participants (26% of total trial enrollment). Here, we report our single institution experience participating in the PATHFINDER study and provide interesting case reports. We aim to illustrate the clinical scenarios and describe potential challenges and considerations in interpreting MCED test results to provide insights into its role in the future of cancer care.

## Materials/methods

Under IRB approval, a retrospective chart review of participants enrolled in the PATHFINDER study was performed. The MCED test used in this case series is based on targeted methylation analysis of cell-free DNA in peripheral blood, using next general sequencing to identify methylation patterns characteristic of cancer and predictive of cancer types. A machine learning classifier determines the presence of a cancer signal and predicts the likely cancer signal origin (CSO), guiding further diagnostic evaluation (4). Cancer risk factors, outcomes of tests and studies performed due to MCED signal positive, time to diagnostic resolution, and treatment outcomes were obtained from chart-review. Cases were selected to illustrate a range of clinical scenarios encountered during the early implementation of MCED testing at our institution.

## Results

From January 2020 to December 2020, our institution enrolled 1735 participants (26% of total study enrollment), 27 of which returned a signal positive for cancer suspicion (1.6%), and ultimately 12 diagnosed cancers (true positives) for a positive predictive value of 44% (**Table 1**). Two of 12 were recurrent cancers in participants > 3 years from cancer therapy. There were 15 signal positives without cancer diagnoses (false positives, **Table 2**). Six of 15 false positive results correlated to monoclonal B-cell lymphocytosis (CLL precursor). During the course of 12-month follow-up for signal negatives (n=1708 participants), 19 were diagnosed with a cancer (site-specific sensitivity: 39%, specificity: 99.1%).

**Table 1:** OHSU PATHFINDER study true positives.

Age	Sex	Prior cancer history or risk factors	Top signal allocation	Final diagnosis
61	F	Breast Cancer	Breast	Breast Cancer, Recurrent; Stage 4
72	F	Breast Cancer	Breast	Breast Cancer, Recurrent; Stage 4
89	F	Thymus cancer, Former smoker	Colon	Colon Cancer; Staging deferred
83	F	No Cancer History	Upper GI	Colon Cancer; Staging deferred
72	F	Breast Cancer	Sarcoma	Dedifferentiated Chondrosarcoma
60	F	No Cancer History	Lymphoid	Follicular Lymphoma; Grade 1-2
74	F	No Cancer History	Lymphoid	Hodgkin Lymphoma; Stage 1a
69	M	Tonsil Cancer	Lymphoid	B-Cell NHL; Stage 4
71	M	Basal Cell Carcinoma, Current smoker	Lymphoid	Follicular Lymphoma; Grade 1-2
58	M	Former smoker	Head and Neck	Oropharyngeal Cancer; Stage 1
79	M	Leukemia	Indeterminate	Prostate Cancer; Stage 2, Relapsed AML
62	M	No Cancer History	Plasma Cell	Macroglobulinemia; SWM

NHL, Non-Hodgkins Lymphoma; SWM, Smoldering Waldenstrom’s Macroglobulinemia; AML, Acute Myeloid Leukemia.

**Table 2:** OHSU PATHFINDER study false positives.

Age	Sex	Prior cancer history or risk factors	Top signal allocation	Final diagnosis
78	M	SCC, BCC of the skin, Current smoker	Lymphoid	Monoclonal B-cell lymphocytosis
71	F	No Cancer History	Lymphoid	Monoclonal B-cell lymphocytosis
70	M	Prostate Cancer	Lymphoid	Monoclonal B-cell lymphocytosis
76	M	Prostate Cancer	Lymphoid	Monoclonal B-cell lymphocytosis
70	M	No Cancer History	Lymphoid	Monoclonal B-cell lymphocytosis
68	F	No Cancer History	Lymphoid	Monoclonal B-cell lymphocytosis
75	M	No Cancer History	Lymphoid	No Cancer Diagnosis
77	F	No Cancer History	Breast	No Cancer Diagnosis
83	F	No Cancer History	Lung	No Cancer Diagnosis
68	F	No Cancer History	Uterus	No Cancer Diagnosis
55	F	No Cancer History	Kidney	No Cancer Diagnosis
62	F	No Cancer History	Breast	No Cancer Diagnosis

SCC, Squamous Cell Carcinoma; BCC, Basal Cell Carcinoma.

### Case #1, true positive, Stage I Hodgkins lymphoma

**Figure 1:**
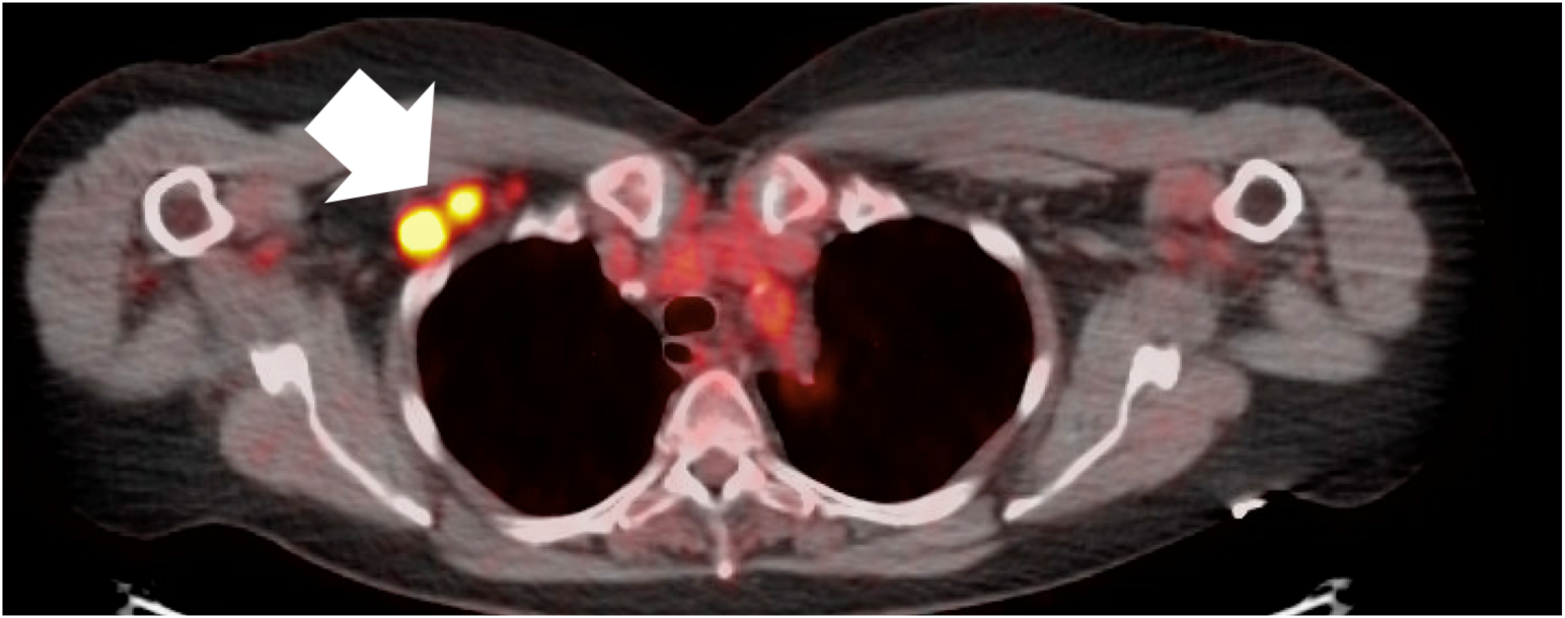
FDG-avid right axillary lymph nodes, biopsy-proven Stage I Hodgkin lymphoma.

A 74-year-old female with past medical history of ANCA vasculitis, who was asymptomatic at the time of testing, had a signal positive result with cancer signal origin consistent with a lymphoid malignancy. She was referred for a PET scan which showed an FDG-avid 1.8 x 1.6 cm axillary lymph node and biopsy (**Figure 1**) was consistent with Stage I (early-stage favorable-risk) classical Hodgkins’s lymphoma. She achieved diagnostic resolution within 3 months and ultimately received combined modality therapy with 2 cycles of adriamycin, bleomycin, vinblastine and dacarbazine chemotherapy and involved-site radiation therapy. She remains in complete remission 4 years following therapy.

### Case #2, true positive, Stage I HPV-related head and neck squamous cell carcinoma

A 58-yo-male with a history of smoking and drinking had MCED testing with a signal positive result with cancer signal origin of head and neck. The patient noted a right cervical lymph node without dysphagia or constitutional symptoms. Ultrasound revealed multiple enlarged right cervical lymph nodes. CT scan showed a 22 mm enhancing right base of tongue mass, as well as enlarged ipsilateral cervical lymph nodes measuring 24 mm in maximal dimension. Fine needle aspiration of a right cervical lymph node revealed p16 positive nonkeratinized squamous cell carcinoma. No distant metastases were noted on PET/CT. He received a formal diagnosis within 3 weeks of initial MCED testing. He received definitive chemoradiation for a Stage I (cT2cN1cM0) p16 positive squamous cell carcinoma of right tonsil/right lateral oropharyngeal wall with right sided cervical lymphadenopathy. He had a complete clinical response to therapy and continues to have no evidence of disease 4 years following therapy.

### Case #3, true positive, Stage 2 prostate cancer and relapsed acute myeloid leukemia

A 79-year-old male with a history of acute myeloid leukemia, asymptomatic and in remission following allogeneic stem cell transplant 6 years prior had a signal positive result with an indeterminate cancer signal origin. Given his age and sex, PSA was obtained and was elevated at 17.3 ng/ml. Urologic oncology performed a transrectal ultrasound-guided biopsy which showed a Stage 2 (cT2N0M0) prostatic adenocarcinoma with Gleason grade 4 + 3 in 5 out of 8 cores. Follow up FDG PET scan showed an ill-defined hypermetabolic osseous lesion along the left lateral aspect of the L4 and L5 vertebrae surrounding the intervertebral space but no other evidence of avid disease. Further evaluation included a core needle biopsy of the L4/L5 lesion which was positive for myeloid leukemia cells and negative for adenocarcinoma. He then underwent treatment with external beam radiation therapy for his isolated and relapsed myeloid leukemia at L4/L5 (24 Gy in 12 fractions) and localized prostate adenocarcinoma (79.2 Gy in 44 fractions) with 16 total months of androgen deprivation therapy and continues to have no evidence of disease from either his prostate cancer or AML 4 years following therapy.

### Case #4, false positive, three cancer signal origins provided, no cancer detected

An 83-year-old female with a history of multiple cutaneous squamous cell carcinomas had a signal positive result with top cancer signal origin of lung. Additional cancer signal origins detected included breast and head and neck. Her workup lasted 3 months and included a chest CT which showed multiple enlarged right axillary and retro-pectoral lymph nodes concerning for possible underlying malignancy. PET/CT confirmed hypermetabolic right axillary and subpectoral lymph nodes with no other PET-avid lesions concerning for malignancy. Biopsy of right axillary lymph nodes was negative. The patient underwent mammography which showed heterogeneously dense breasts possibly obscuring small masses. Therefore, the patient underwent breast MRI which showed no evidence of breast malignancy. After negative workup 3 months following enrollment date, patient and provider were amenable to halting further investigations due to no cancer diagnosis being found. The patient underwent follow up PET/CT 1 year later which showed no evidence of metabolically active disease, and the patient continues to have no cancer diagnosis 4 years after initial workup.

### Case #5, true positive, colon cancer not completely staged nor treated

A 89-year-old woman with past medical history of thymoma with myasthenic gravis on chronic prednisone, status post thymectomy and thoracic radiation 12 years prior, had a signal positive result with cancer signal origin of colon. Due to cardiac history, she underwent CT colonography in place of endoscopy, which identified multifocal large polyps in multiple segments of the colon which were highly suspicious for a colon malignancy. She was referred to surgery, who recommended that she undergo colonoscopy to determine the best treatment path forward. Colonoscopy showed marked diverticulosis involving 6mm polyps in the sigmoid colon, and 5mm polyps in the descending colon and hepatic flexure, from which samples were obtained for biopsy. Biopsy results were inconclusive, and the surgeon recommended laparoscopic right colectomy. Ultimately, the patient chose not to undergo surgical resection and elected for palliative care due to her comorbidities.

## Discussion

Our single-institution experience highlights one of the first comprehensive applications of multi-cancer early detection testing within the framework of the PATHFINDER study, offering key insights into clinical utility, diagnostic challenges, and patient impact. Through the evaluation of 27 signal positive patient cases, we observed a spectrum of outcomes, illustrating key aspects of MCED testing’s potential integration into clinical care, underscoring potential benefits of early detection, and highlighting challenges of interpreting results.

There is a paucity of literature detailing patient-specific pathways for those with an MCED signal positive test (3, 5). Among our cases was a true positive detection of stage 2 prostate cancer and relapsed acute myeloid leukemia after bone marrow transplant. Given the early-stage diagnosis in a clinically asymptomatic patient, the patient underwent successful and localized radiation with no evidence of disease for 4 years following treatment. This case exemplifies the power of MCED testing in its ability to detect subclinical relapse before overt clinical symptoms or major hematologic abnormalities. Our two additional true positive cases with stage I diagnoses of Hodgkins Lymphoma and HPV-related head and neck squamous cell carcinoma demonstrate the value of early detection with early intervention leading to patients experiencing fewer complications and maintaining a longer period of disease control.

While MCED testing identified true positive cases leading to successful early intervention, one true positive detection of colon cancer in a 90-year-old woman with complex past medical history raised ethical and clinical considerations regarding screening in patients who, even if diagnosed with cancer, may not be candidates for definitive treatment. In this patient, significant comorbidities and advanced age yielded challenges in aggressive therapy and they ultimately chose not to proceed with curative treatment. Another particularly challenging case involved a false positive for an 83-year-old female who underwent extensive workup, including CT, PET-CT, biopsy, mammography, and a breast MRI. Her workup was complicated by the multiple cancer signal origin signals provided, and while no malignancy was found, the psychological distress and financial impact is non-trivial. These types of challenges with medical workup and psychosocial distress from diagnostic workups have been previously reported (6, 7).

Overall, our case series contributes to the growing body of literature on MCED implementation by providing a nuanced perspective on potential benefits and considerations. Future research should focus on establishing clear clinical pathways to manage indeterminate or false-positive results. As MCED testing continues to evolve, its integration into routine cancer screening will require careful calibration to maximize benefits while minimizing potential harms.

## Data Availability

The original contributions presented in the study are included in the article/supplementary material. Further inquiries can be directed to the corresponding author.
